# A Network of Networks Perspective on Global Trade

**DOI:** 10.1371/journal.pone.0133310

**Published:** 2015-07-21

**Authors:** Julian Maluck, Reik V. Donner

**Affiliations:** 1 Potsdam Institute for Climate Impact Research, Potsdam, Germany; 2 Department of Physics, Humboldt University, Berlin, Germany; Tianjin University, CHINA

## Abstract

Mutually intertwined supply chains in contemporary economy result in a complex network of trade relationships with a highly non-trivial topology that varies with time. In order to understand the complex interrelationships among different countries and economic sectors, as well as their dynamics, a holistic view on the underlying structural properties of this network is necessary. This study employs multi-regional input-output data to decompose 186 national economies into 26 industry sectors and utilizes the approach of interdependent networks to analyze the substructure of the resulting international trade network for the years 1990–2011. The partition of the network into national economies is observed to be compatible with the notion of communities in the sense of complex network theory. By studying internal versus cross-subgraph contributions to established complex network metrics, new insights into the architecture of global trade are obtained, which allow to identify key elements of global economy. Specifically, financial services and business activities dominate domestic trade whereas electrical and machinery industries dominate foreign trade. In order to further specify each national sector’s role individually, (cross-)clustering coefficients and cross-betweenness are obtained for different pairs of subgraphs. The corresponding analysis reveals that specific industrial sectors tend to favor distinct directionality patterns and that the cross-clustering coefficient for geographically close country pairs is remarkably high, indicating that spatial factors are still of paramount importance for the organization of trade patterns in modern economy. Regarding the evolution of the trade network’s substructure, globalization is well-expressed by trends of several structural characteristics (e.g., link density and node strength) in the interacting network framework. Extreme events, such as the financial crisis 2008/2009, are manifested as anomalies superimposed to these trends. The marked reorganization of trade patterns, associated with this economic crisis in comparison to “normal” annual fluctuations in the network structure is traced and quantified by a new widely applicable generalization of the Hamming distance to weighted networks.

## Introduction

In the last years, the international trade network (ITN, also often referred to as the world trade web) has caught rising attention among the scientific community. To this date, there have been numerous studies on the topological properties of the ITN which is commonly defined based upon the evolving import/export relationships between countries [1–10]. The ITN has been analyzed as both binary and weighted, as well as directed and undirected complex network. Previous studies emphasize specific characteristics of the ITN, such as the distinctive non-random topology of world trade [1] or the exposure of a core-periphery structure among countries [11]. More detailed analyses have focused on commodity-specific multi-network approaches [12, 13], also addressing important aspects such as the community structure of the ITN [14, 15]. Recent findings also shed light on the roles and functions of individual countries in the ITN, highlighting the decline of the Western dominance in global trade [16].

Previous research on the ITN has mostly treated the countries as single nodes in the network. This approach neglects important substructures of the national economies. With the availability of multi-regional input-output (MRIO) tables [17–19] valuable and novel insights into the substructure of the ITN can be obtained. Here, each national economy is decomposed into industrial sectors trading with each other both domestically and internationally. Interpreted as a directed and weighted network, MRIO tables provide a more complete and highly resolved picture of the ITN based on monetary flows between industries. Interdependent networks exhibit specific characteristics in the propagation of shocks different from those of single, non-interacting networks [20, 21]. Therefore, an investigation of the ITN’s substructure is vital in order to better understand the underlying risks of the spreading of an economic crisis that may be triggered by a node’s failure to produce its standard output [22, 23].

This refinement of the ITN allows for a more holistic view on global trade and on the complex interdependencies within the present-day global economy. In the process of globalization, trade patterns have been reorganizing and international trade has been increasing almost continuously [24, 25]. In this setting the following questions arise naturally when investigating the topological structure of world trade: How meaningful is the notion of national economies in an international globalized economy, where few transnational corporations hold dominant positions on a global scale [26]? What roles do specific industrial sectors and countries play in the ITN? In which industries and nations have trade relationships reorganized most along with globalization? How do national economies adapt to increasing foreign trade relations?

**Fig 1:**
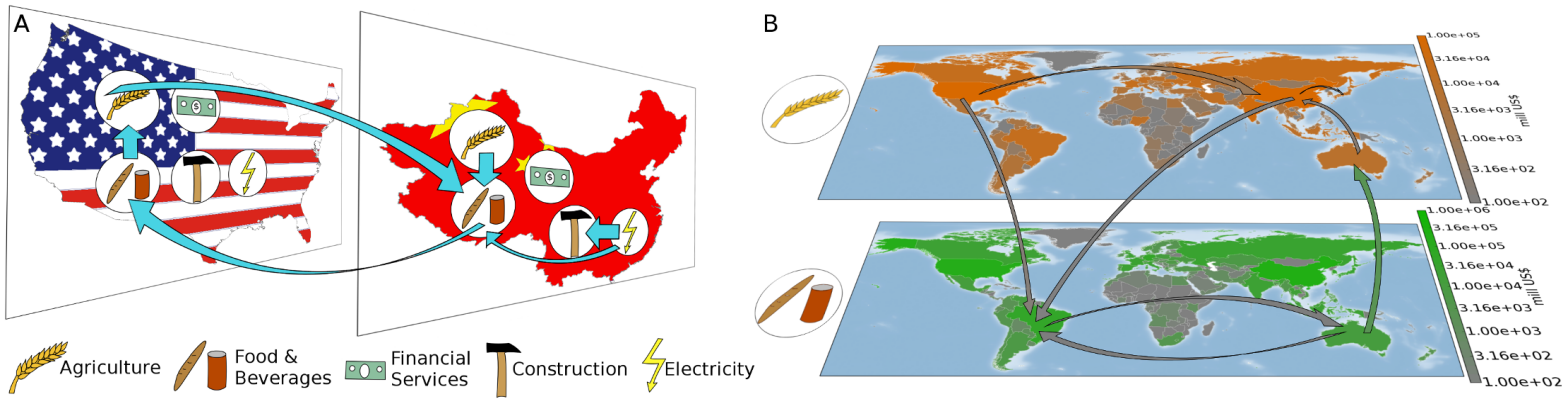
Illustration of sample subgraphs in the ITN. (A) According to the national partition 𝓒_*c*_ and (B) according to the sectoral partition 𝓒_*s*_.

In this work we illustrate that analyses of MRIO tables by means of complex network theory offer meaningful techniques to address these questions. For this purpose, we employ a MRIO database comprising annually averaged monetary flows between 186 countries with 26 industrial sectors for the years 1990–2011. In particular, we focus on the interpretation of the ITN as a network of mutually interdependent subnetworks. As each node in this network is labeled with its country and industry, nodes can be intuitively grouped together either by country or by industrial sector, building a national and sectoral partition, respectively (see Fig 1). In addition, we determine further data-driven partitions by utilizing established community detection algorithms [27]. We measure the modularity score [28] to assess the quality of a partition with respect to the notion of a community in a network and quantify the similarity between two partitions of the same network via the variation of information [29]. With the definition of partitions of the ITN, network measures can be distinguished into internal and cross-subgraph measures [30].

We utilize the aforementioned approach to identify key players with respect to specific trade patterns and the assignment of roles to nodes in the ITN. For this purpose, we consider proper generalizations of standard network measures (node strength, clustering coefficient and betweenness) to interdependent networks. To address the implications of the globalization process for the resulting network structure, the evolution of the ITN structure is traced for the period 1990–2011. We quantify trends in the respective network measures relating to internal or cross-linkages of subgraphs. The appearance of significant anomalies from these trends suggest the existence of an extreme event corresponding to the global financial crisis in 2008/2009. We further introduce a generalization of the Hamming distance to weighted networks as a measure to quantify the inter-annual reorganization of trade patterns and illustrate its effectiveness in recognizing large-scale economic shocks and crises. Our results illustrate that the interpretation of the ITN as a network of networks exhibits new insights into the structural backbone of global trade and offers appropriate tools for the investigation of cross-sectoral economic relations at both the global and regional scale.

## Materials and Methods

### Data and network construction

MRIO data summarize the monetary flows between industrial sectors and can be meaningfully interpreted as a weighted and directed network of interdependent subgraphs, where nodes correspond to sectors, weighted and directed links describe the annual volume of financial flows, and subgraphs can be associated with national economies or the same industry sectors across the ITN (cf. Fig 1).

In this work, we utilize data from the Eora MRIO table providing annual data for 1990–2011 [18, 31]. The Eora database collects highly resolved trade data, decomposing each of the 186 countries contained into 26 industrial sectors. Monetary flows between two industrial sectors are given in nominal US $. Thus, we construct for each year a network with *N* = 4836 nodes. For the ITN in 1990, we consider two nodes to be connected if the monetary flow between two nodes exceeds 1 million US $, assuming that smaller values primarily represent artifacts from harmonization procedures during the compilation process of Eora [23]. To minimize inflationary effects, we adapt the threshold to the yearly US inflation rate [32] in the construction of the ITN for the following years. After the establishment of links, a weight proportional to the monetary flow is then attributed to each edge. In order to distinguish structural changes from effects arising from inflation, we normalize the weights to the annual global trade volume for each year.

**Fig 2:**
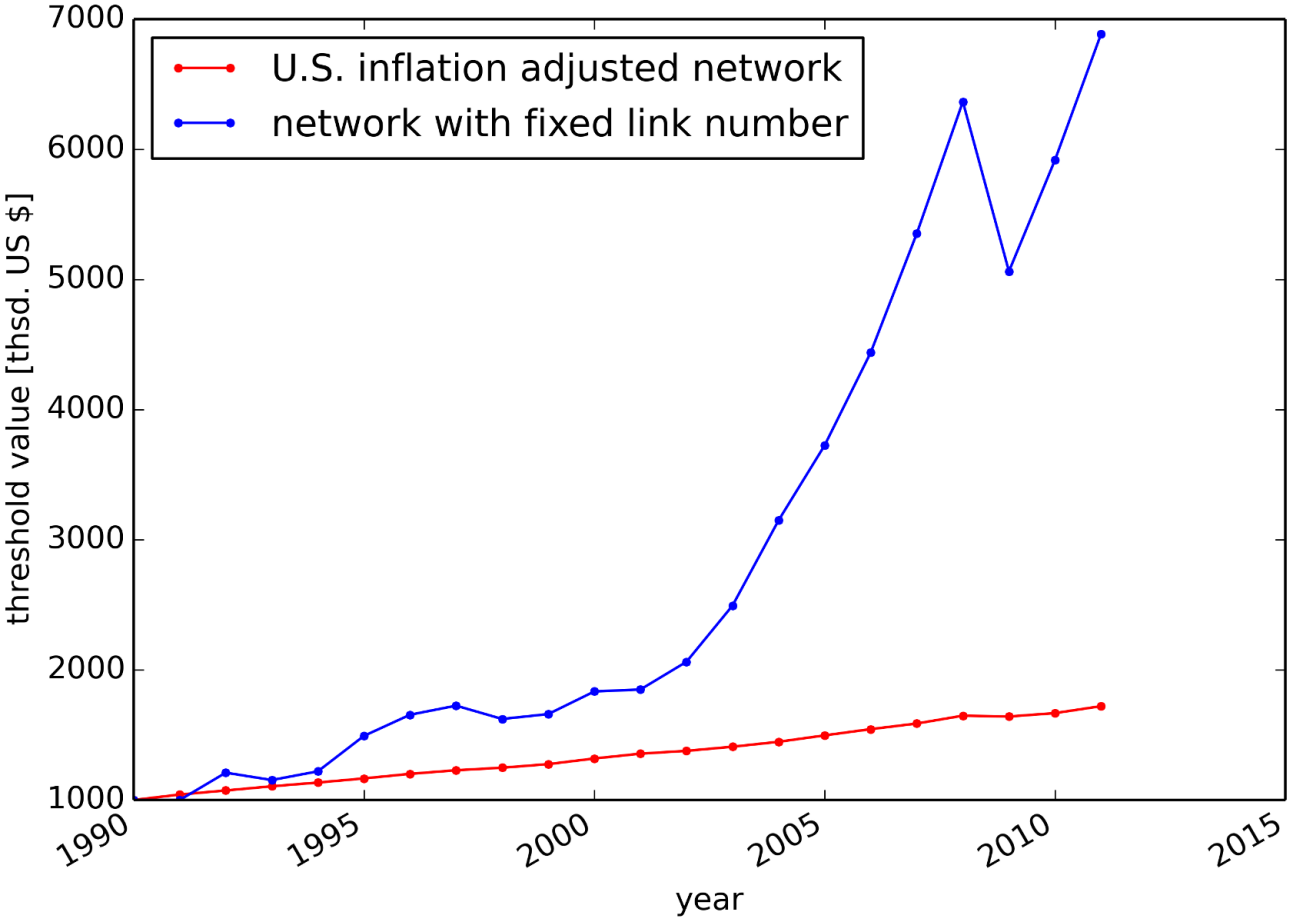
Thresholds for network construction. Lowest weight in the ITN taking US inflation into account (red) and in the network with a fixed number of links (blue).

We further construct for each year a second network by fixing the amount of links to the number of links in the ITN in 1990. Thereby, we assess the robustness of the results with respect to varying the threshold during the network construction and disentangle effects which are very sensitive to threshold variations in the construction process. The utilized threshold values of the ITN and the network with constant link density are shown in Fig 2. The trend in the threshold value for the network with constant link density implies a rise in trade volume in US $ and increasing entanglement in trade relationships. It should be noted that values of monetary flows in US $ cannot be mapped trivially to the physical trade of goods. For example, a constant flow of goods could result in a non-constant monetary flow, given volatile price and exchange rate fluctuations. However, the mapping of monetary flows to physical flows is far from trivial [33] and beyond the scope of this work.

### Subnetworks

In order to analyze the substructure of the ITN, we interpret it as a network of interdependent subnetworks [30, 34]. For this purpose, we assume a network *G* = (*V*, *E*) that consists of the set of nodes *V* and set of links *E*, with the number of nodes *N* = ∣*V*∣ and the number of links *m* = ∣*E*∣. The graph can be decomposed into subgraphs $G_{{}^{{}_{p}}}^{\prime }[V_{{}^{{}_{p}}}^{\prime }]$ that are induced by the node subset $V_{p}^{\prime }\subset V$ with $\cup_{p}V_{p}^{\prime }$ and $V_{{}^{{}_{p}}}^{\prime }\cap^{}V_{{}^{{}_{q}}}^{\prime }=\emptyset$. Links can be distinguished according to whether or not they connect nodes in the same subgraph, i.e. the (internal) link sets $E_{{}^{{}_{pp}}}^{\prime }$ connect nodes belonging to the same subgraph *p*, whereas cross-link sets $E_{{}^{{}_{p\neq q}}}^{\prime }$ connect subgraphs via nodes belonging to the subgraphs *p* and *q*, respectively. The full graph is represented by the possibly asymmetric adjacency matrix **A**, with *a*_*ij*_: = (**A**)_*ij*_, and the weight matrix **W**, with *w*_*ij*_: = (**W**)_*ij*_ being proportional to the monetary flow between node *i* and *j*. We further define the *N* × *N*-matrices

$${\left(A_{\text{auto}}\right)}_{ij}=\{\begin{array}{cc} 1, & \text{if}\;\left(i,j\right)\in \;\cup_{p}E_{pp}^{\prime }\\ 0, & \text{else} \end{array}\;;\;{\left(A_{\text{cross}}\right)}_{ij}=\{\begin{array}{cc} 1, & \text{if}\;\left(i,j\right)\in \;\cup_{p\neq q}E_{pq}^{\prime }\\ 0, & \text{else} \end{array}\;$$ (1)

that are convenient for the measurement of quantities that describe internal subgraph structure (**A**_auto_) or cross-subgraph relations (**A**_cross_) with **A** = **A**_auto_ + **A**_cross_.

For the ITN, the subsets $V_{{}^{{}_{p}}}^{\prime }$ can be defined in various ways. Each node of the network belongs to a specific country *c* and to an industrial sector *s*. Therefore, one of the self-evident partitions 𝓒_*c*_ is a classification of nodes by country. Fig 1A illustrates an excerpt of this partition, with the full network consisting of 186 national subgraphs containing 26 nodes each. A complementary approach 𝓒_*s*_ is to define subgraphs consisting of nodes from the same industrial sectors, depicted in Fig 1B. The employment of dedicated community detection algorithms [27] provides a third reasonable way to partition the ITN. In complex network theory, a community is characterized by high interconnectedness among the nodes within the same community, whereas linkages to nodes out of the community are sparse.

### Local network measures

#### Node degree and node strength

In an undirected and unweighted network with adjacency matrix **A**′, the connectivity of a node *i* is described by its degree $k_{i}=\underset{j}{\sum }a_{{}^{{}_{ji}}}^{\prime }=\underset{j}{\sum }a_{{}^{{}_{ij}}}^{\prime }$. In the directed case (with adjacency matrix **A**), it is feasible to distinguish the in-degree $k_{i}^{in}$ and the out-degree $k_{i}^{out}$, defined as

$$k_{i}^{in}=\sum_{j=1}^{N}a_{ji}\;;\;k_{i}^{out}=\sum_{j=1}^{N}a_{ij}\;.$$ (2)

In weighted networks, the degree *k*_*i*_ is commonly replaced by the node strength *s*_*i*_, which for undirected networks with weight matrix **W**′ reads $s_{i}=\underset{j}{\sum }w_{{}^{{}_{ij}}}^{\prime }$. In the directed case (with weight matrix **W**), one again distinguishes in- and out-strengths defined as $s_{i}^{in}=\sum_{j}w_{ji}$ ($s_{i}^{out}=\sum_{j}w_{ij}$). With a given partition 𝓒, the strength can be further distinguished into internal strength *s*_*i*;auto_ and cross-strength *s*_*i*;cross_

$$\begin{array}{c} \begin{array}{ccccc} s_{i;\text{auto}}^{in} & = & \sum_{j=1}^{N}w_{ji}{\left(A_{\text{auto}}\right)}_{ji}\;;\;s_{i;\text{cross}}^{in} & = & \sum_{j=1}^{N}w_{ji}{\left(A_{\text{cross}}\right)}_{ji}\;;\\ s_{i;\text{auto}}^{out} & = & \sum_{j=1}^{N}w_{ij}{\left(A_{\text{auto}}\right)}_{ij}\;;\;s_{i;\text{cross}}^{out} & = & \sum_{j=1}^{N}w_{ij}{\left(A_{\text{cross}}\right)}_{ij}\;. \end{array} \end{array}$$ (3)

#### Local clustering coefficient

**Fig 3:**
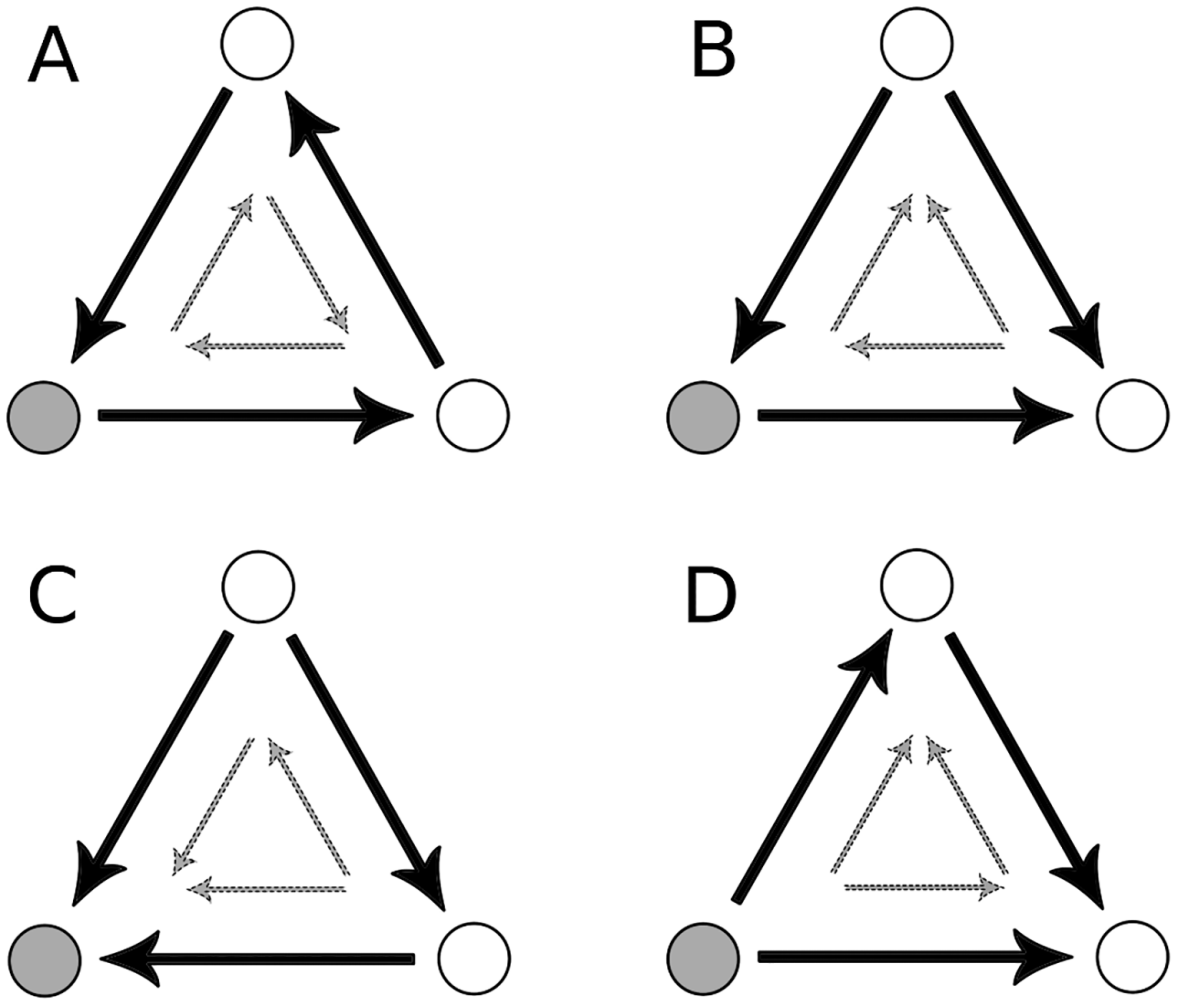
Clustering coefficients in a directed network. Following the definitions in Eq (4) the coefficients (A) $C_{i}^{cyc}$, (B) $C_{i}^{mid}$, (C) $C_{i}^{in}$ and (D) $C_{i}^{out}$ are shown. For each coefficient two motifs contribute which are indicated by the three large black links and the three small gray links, respectively.

The local clustering coefficient measures the probability of the existence of a link between two randomly selected neighbors of node *i*. In directed networks different definitions of clustering coefficients exist. In this work, we follow the classification scheme of Fagiolo [35] and consider the following five clustering coefficients (Fig 3):

$$\begin{array}{c} \begin{array}{c} C_{i}^{cyc}=\frac{{\left({\hat{W}}^{3}\right)}_{ii}}{k_{i}^{in}k_{i}^{out}-k_{i}^{↔}}\;;\;C_{i}^{mid}=\frac{{\left(\hat{W}{\hat{W}}^{T}\hat{W}\right)}_{ii}}{k_{i}^{in}k_{i}^{out}-k_{i}^{↔}}\;;\;\\ C_{i}^{in}=\frac{{\left({\hat{W}}^{T}{\hat{W}}^{2}\right)}_{ii}}{k_{i}^{in}\left(k_{i}^{in}-1\right)}\;;\;C_{i}^{out}=\frac{{\left({\hat{W}}^{2}{\hat{W}}^{T}\right)}_{ii}^{3}}{k_{i}^{out}\left(k_{i}^{out}-1\right)}\;;\;\\ C_{i}^{all}=\frac{{\left(\hat{W}+{\hat{W}}^{T}\right)}^{3}}{\left(k_{i}^{in}+k_{i}^{out}\right)\left(k_{i}^{in}+k_{i}^{out}-1\right)-2k_{i}^{↔}}\;. \end{array} \end{array}$$ (4)

Here $k_{i}^{in}$ represents the in-degree and $k_{i}^{out}$ the out-degree of node *i* according to Eq (2), while $k_{i}^{↔}={\left(A^{2}\right)}_{ii}$ denotes the number of bilateral links associated with *i*. With $\hat{\text{W}}={\text{W}}^{1/3}=\{w_{ij}^{1/3}\}$ the clustering coefficients take weights and directionality patterns into account. The “cycle” pattern $C_{i}^{cyc}$ and “middleman” pattern $C_{i}^{mid}$ in Eq (4) describe a node’s importance as transmitter of monetary flows in the 3-motif, whereas $C_{i}^{in}$ and $C_{i}^{out}$ indicate a node’s role as sink and source, respectively. The coefficient $C_{i}^{all}$ considers all possible 3-motifs.

In order to account for a given partition of a network into different subnetworks, the concept of cross-clustering coefficients has been introduced in [30] and subsequently applied in [36, 37]. For the case of directed networks, one has to again distinguish cross-clustering coefficients according to different patterns. Using

$$\begin{array}{c} {\left({\tilde{W}}^{p}\right)}_{ij}=\{\begin{array}{cc} {\hat{w}}_{ij} & \text{if}\;\left(i,j\right)\in \;E_{pp}^{\prime }\\ 0 & \text{else} \end{array} \end{array}$$ (5)

in networks with a defined subgraph structure and $k_{i}^{p}$ being the number of connections from node *i* to subgraph *p* (the cross-degree of *i* with respect to *p* [30]), the local cross-clustering coefficient for the “cycle” pattern yields:

$$C_{i}^{p;cyc}=\frac{{\left(\hat{W}{\tilde{W}}^{p}\hat{W}\right)}_{ii}}{k_{i}^{p;in}k_{i}^{p;out}-k_{i}^{p;↔}}\;,$$ (6)

with the cross-in-degree $k_{i}^{p;in}=\sum_{j\in V_{{}^{{}_{p}}}^{\prime }}a_{ji}$, cross-out-degree $k_{i}^{p;out}=\underset{j\in V_{p}^{\prime }}{\sum }a_{ij}$ and bilateral cross-degree $k_{i}^{p;↔}=\sum_{j\in V_{{}^{{}_{p}}}^{\prime }}a_{ij}a_{ji}$.

#### Betweenness

The betweenness [38] measures the centrality of a node with respect to its role as a mediator of the flow between nodes in the network. Generalizing the idea to interdependent networks, the cross-betweenness [30] is defined as

$$b_{i}^{pq}=\sum_{j\in V_{p}^{\prime },k\in V_{q}^{\prime };k,j\neq i}\frac{\sigma_{jk}\left(i\right)}{\sigma_{jk}}\;,$$ (7)

quantifying the importance of node *i* to connect subgraphs *p* and *q*. Here, *σ*_*jk*_ is the total number of shortest paths from node *j* to node *k*, while *σ*_*jk*_(*i*) is the number of these paths that include node *i*. To calculate shortest paths, we neglect the weight information of links, focusing on the question whether trade relations between sectors have been established or not. Note, that shortest paths between nodes in $V_{{}^{{}_{p}}}^{\prime }$ and $V_{{}^{q}}^{\prime }$ may pass through a third subgraph.

### Global network measures

#### Link density

The ratio between the existing number of links and the maximum number of possible links among the considered set of nodes is referred to as the link density. Consequently, in the full directed network the link density reads *ρ*_full_ = *m*/*N* ^2^, given that self-connections are considered during network construction (*ρ*_full_ = *m*/*N*(*N* − 1) if self-connections are neglected). In a network of interdependent networks further topological properties are revealed by distinguishing between the internal link density and the cross-edge density:

$$\rho_{\text{auto}}=\frac{\sum_{p}\left|E_{pp}^{\prime }\right|}{\sum_{p}{\left|V_{p}^{\prime }\right|}^{2}}\;;\;\rho_{\text{cross}}=\frac{\sum_{p\neq q}\left|E_{pq}^{\prime }\right|}{\sum_{p\neq q}|V_{p}^{\prime }\left|\right|V_{q}^{\prime }|}\;.$$ (8)

In partitions with subnetworks that are determined by community detection algorithms and result in a high modularity score, *ρ*_auto_ exceeds *ρ*_cross_ by definition.

#### Global cross-clustering coefficient

In order to assess the structure of triangular linking patterns between subnetworks, a global perspective on the cross-clustering coefficient is required. With the local cross-clustering coefficient $C_{{}_{i}}^{{}^{p;cyc}}$ of node *i* (cf. Eq (6)), the associated global cross-clustering coefficient from partition *p* to partition *q* is therefore defined as [30, 36]

$$C_{q}^{p;cyc}:=\sum_{i\in V_{q}^{\prime }}C_{i}^{p;cyc}\;.$$ (9)

Note that the relation in Eq (9) is not symmetric, i.e. $C_{q}^{p}\neq C_{p}^{q}$. The same approach can be applied to all directionality patterns introduced in Eq (4).

#### Reciprocity

In a directed network the reciprocity characterizes the probability that a randomly chosen link between two nodes also exists in the opposite direction. As self-connections do not provide additional information about this probability, flows of the node to itself are excluded:

$$r=\frac{1}{m}\mathrm{Tr}{\left[A-\text{diag}(A)\right]}^{2}\;.$$ (10)

The respective inner and cross-reciprocity is then obtained by

$$r_{\text{auto}}=\frac{1}{|A_{\text{auto}}|}\mathrm{Tr}{\left[A_{\text{auto}}-\text{diag}\left(A_{\text{auto}}\right)\right]}^{2}\;;\;r_{\text{cross}}=\frac{1}{|A_{\text{cross}}|}\mathrm{Tr}{\left[A_{\text{cross}}\right]}^{2}\;.$$ (11)

#### Hamming distance

The Hamming distance quantifies the dissimilarity between two networks *G*, G* that have the same set of nodes. Originally designed for unweighted networks [39] with

$$H\left(G,G^{*}\right)=\frac{\sum_{ij}\left|a_{ij}-a_{ij}^{*}\right|}{N^{2}}\;,$$ (12)

the principle is extendable to weighted networks. Here, we introduce the following generalizations and compare their performance:

$$H_{s}\left(G,G^{*}\right)=\frac{1}{N^{2}}\sum_{ij}\frac{|w_{ij}-w_{ij}^{*}|}{w_{ij}+w_{ij}^{*}}\;;$$ (13)

$$H_{m}\left(G,G^{*}\right)=\frac{1}{N^{2}}\sum_{ij}\frac{|w_{ij}-w_{ij}^{*}|}{\text{max}(w_{ij},w_{ij}^{*})}\;;$$ (14)

$$H_{a}\left(G,G^{*}\right)=\frac{1}{N^{2}}\sum_{ij}\frac{|w_{ij}-w_{ij}^{*}|}{\Phi }\;\;\text{with}\;\Phi =\frac{\sum_{ij}w_{ij}+\sum_{ij}w_{ij}^{*}}{\left|w|+\right|w^{*}|}\;.$$ (15)

In *H*_*a*_ the differences of link weights are normalized with respect to the average weight per link in the two networks. The measure is dominated by links with large weight differences, whereas this effect is balanced in the definitions of *H*_s_ and *H*_m_. More specifically, each summand (i.e. pair of nodes) in Eqs (13) and (14) accounts for a value in the interval [0, 1]. A summand is 1 if a link from node *i* to *j* is present in *G* and absent in G* (or vice versa). Therefore, *H*_s_ and *H*_m_ can be considered as an extension of Eq (12) by additionally considering links that are present in both networks but have different weights. We can thus distinguish between different contributions to the Hamming distance according to Radebach et al. [40], allowing for a more detailed assessment of the dissimilarity between the two networks. Let *b* and *c* be the number of pairs that are linked in one network and unconnected in the other, with *b* counting the links in the network with higher link density *ρ*. Then, the Hamming distance *H*_*m*_ can be decomposed as follows:

$$H_{m}\left(G,G^{*}\right)=\Delta \rho +l_{b}+\Delta w_{m}:=\frac{b-c}{N^{2}}+\frac{2c}{N^{2}}+\frac{1}{N^{2}}\sum_{ij}\frac{|w_{ij}-w_{ij}^{*}|a_{ij}a_{ij}^{*}}{\text{max}(w_{ij},w_{ij}^{*})}\;.$$ (16)

Thus, three summands describing specific structural differences contribute to the Hamming distance: the link density difference Δ*ρ* = (*b* − *c*)/*N* ^2^, the blinking links *l*_*b*_ = (2*c*)/*N* ^2^ [41, 42], and Δ*w*_*m*_ summarizing the change in weights between pairs where both networks exhibit a link. The “corrected” Hamming distance is defined by neglecting contributions arising from link density difference: $H_{m}^{*}=H_{m}-\Delta \rho$. The definitions Δ*w*_*s*_ and $H_{s}^{*}=H_{s}-\Delta \rho$ can be adapted analogously.

### Comparison of partitions

#### Modularity

One measure for the quality of a partitioning is modularity [28]. The modularity *Q* is defined by the difference between the actual number of links within a community and the number that would be expected in a randomly linked network with the same degree sequence. For an undirected and unweighted network with adjacency matrix **A**′ the modularity is defined as [28]

$$Q_{u}=\frac{1}{2m}\sum_{ij}(a_{ij}^{\prime }-\frac{k_{i}k_{j}}{2m})\delta \left(S_{i},S_{j}\right)\;,$$ (17)

where *k*_*i*_ is the degree of node *i* and *S*_*i*_, *S*_*j*_ denote the indices of the communities that nodes *i* and *j* belong to. The Kronecker delta *δ*(*S*_*i*_, *S*_*j*_) assures that only node pairs within the same community contribute to the sum in Eq (17).

Although various generalizations of the modularity exist, there is less consensus about the formulation of a generally applicable quality function for partitions in directed networks [43]. Arenas et al. [44] proposed defining the modularity in directed networks as

$$Q_{d}=\frac{1}{m}\sum_{ij}(a_{ij}-\frac{k_{i}^{out}k_{j}^{in}}{m})\delta \left(S_{i},S_{j}\right)\;,$$ (18)

comparing the link distribution within a community to the expectation in the directed configuration model [45], with $k_{i}^{in(out)}$ as defined in Eq (2). As suggested by Kim et al. [46] this approach does not fully account for the directionality of links between nodes with the same in- and out-degree, respectively. Alternative definitions for modularity are based on the attributes of links with respect to the probability density of a random walker in the network [43]. However, in the context of international trade, these definitions would lead to misleading interpretations that arise due to the fact that industry sectors produce added value and request final demand, leading to unconserved monetary flows in the trade network. We therefore utilize Eqs (17) and (18) for this work. By replacing the degree $k_{i}^{in}$ ($k_{i}^{out}$) by the strength $s_{i}^{in}$ ($s_{i}^{out}$) and the number of links *m* with the sum of weights ∣**W**∣ = ∑_*ij*_ *w*_*ij*_ in the network, the definitions of *Q* given above are also applicable to weighted networks, i.e.

$$Q_{d,w}=\frac{1}{\left|W\right|}\sum_{ij}(w_{ij}-\frac{s_{i}^{out}s_{j}^{in}}{\left|W\right|})\delta \left(S_{i},S_{j}\right)\;.$$ (19)

#### Variation of information

In order to quantify the difference between two partitions, we measure the variation of information [29]

$$VI\left(C,C^{\prime }\right)=-\sum_{q=1}^{n_{q}}P\left(q\right)\log P\left(q\right)-\sum_{q^{\prime }=1}^{n_{q}^{\prime }}P\left(q^{\prime }\right)\log P\left(q^{\prime }\right)-2\sum_{q,q^{\prime }}P\left(q,q^{\prime }\right)\log\frac{P(q,q^{\prime })}{P\left(q\right)P\left(q^{\prime }\right)}\;.$$ (20)

Here, the probability that a randomly drawn node belongs to cluster *q* in partition 𝓒 with *n*_*q*_ clusters is denoted by *P*(*q*). *P*(*q*, *q*′) is then the joint probability that a random node belongs to *q* in 𝓒 and to *q*′ in 𝓒′. The value of VI returns 0 if 𝓒 = 𝓒′ and reaches its maximum value of log*N* in the case of *n*_*q*_ = *N* and *n*_*q*′_ = 1.

## Results

As previous studies have exposed, trade between countries exhibits a highly non-trivial topology [2, 5–7]. Trade networks in present-day globalized economy are becoming increasingly complex, resulting in interwoven trade activity between national economies and between industrial sectors.

### Subnetworks & Communities

An evident question in contemporary interconnected global economy is how meaningful the notion of a national economy still is. We address this question by comparing the network topology of a national partition (𝓒_*c*_) with the topology of the complementary sectoral partition (𝓒_*s*_). A priori both partitions have their own justification. On the one hand, domestic (internal) trade within a country is supported by a common policy framework and short geographical distances. Thus, transportation and transaction costs between sectors in the same country are kept comparatively low. On the other hand, in the industry classification used for this study, many companies that are part of the supply chain of one product are aggregated to the same industrial sector. Therefore, we expect that for a multi-level production process of goods, complex supply chains result in high trading activity within the same sector.

We assess how the definition of the national partition 𝓒_*c*_ and the sectoral partition 𝓒_*s*_ coincides with the notion of communities in network theory [47]. For the community detection we first consider the undirected and unweighted definition (Eq (17)) and utilize a distinguished community detection algorithm and compare its performance with 𝓒_*c*_ and 𝓒_*s*_. Specifically, we employ the “multilevel algorithm” developed by Blondel et al., that extracts communities by a heuristic method based on modularity optimization [27], and results in the partition 𝓒_*m*_. The algorithm was tested to return a relatively high modularity at fast calculation time compared to other algorithms.

Two examples of communities of the partition 𝓒_*m*_ in the ITN of the year 2005 are listed in Table 1. We find that the “multilevel algorithm” preferably assigns nodes belonging to the same country also to the same community. Furthermore, strong economic interdependence resulting from geographical proximity or historical and political connections are represented in the community structure, e.g. most industries of France and Algeria are assigned to the same community. This example illustrates that the communities found by the multilevel algorithm tend to follow the national partition rather than the sectoral one.

**Table 1:** Compositions of two selected communities in 𝓒_*m*_ in the ITN 2005.

Community A		Community B	
**# nodes**	**country**	**# nodes**	**country**
23	Germany	24	France
26	Austria	25	Algeria
24	Switzerland	1	Germany
20	Czech Republic	1	Belgium
25	Hungary	1	Luxembourg
24	Slovakia	1	Mauritania
24	Slovenia	1	Czech Republic
5	Denmark	-	-
3	Russia	-	-
2	Netherlands	-	-
2	Belgium	-	-
2	Poland	-	-
2	UK	-	-
2	Italy	-	-
2	Sweden	-	-
2	Lithuania	-	-
2	Finland	-	-
2	Norway	-	-
2	Turkey	-	-
+ 16 countries with 1 node each		-	-

**Fig 4:**
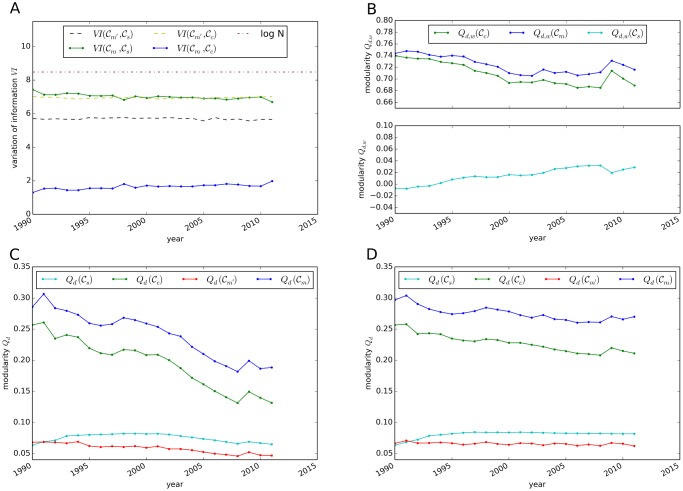
Topological properties of different partitions of the ITN. (A) Evolution of the variation of information (*VI*) as similarity measure between two partitions of the ITN for 1990–2011. (B) Modularity *Q*_*d*, *w*_ in the directed, weighted network for the partitions 𝓒_*c*_, 𝓒_*s*_ and 𝓒_*m*_ in the ITN. (C) Unweighted modularity *Q*_*d*_ for the partitions 𝓒_*c*_, 𝓒_*s*_, 𝓒_*m*_ in the ITN and for 𝓒_*m*′_ in the random graph with identical degree distributions. (D) Unweighted modularity *Q*_*d*_ for partitions in the ITN with constant link density.

To further quantify this finding, we measure the variation of information (VI) (Eq (20)) for the ITN for the years 1990–2011 (see Fig 4A). For all years, the national partitions 𝓒_*c*_ show the highest similarity with the partition of highest modularity, 𝓒_*m*_. Thus, a comparison between these two partitions allows for an identification of the strongest international trade relationships forming the backbone of global trade. To assess the significance of the similarity between 𝓒_*c*_ and 𝓒_*m*_, we compare the values of VI with those computed for the partition 𝓒_*m*′_ which is obtained from a typical representation of the configuration model [48], i.e. from a random graph that obeys the same degree sequence as the original ITN. As expected, the partition 𝓒_*m*′_ differs significantly from 𝓒_*c*_, as links are drawn at random in the configuration model. However, this behavior is not observed for the sectoral partition. In fact, as *VI*(𝓒_*s*_, 𝓒_*m*_) > *VI*(𝓒_*s*_, 𝓒_*m*′_), we conclude that 𝓒_*s*_ does not exhibit the features that are expected for communities in the traditional network theoretical sense. Therefore, our results indicate that international trade relationships are not primarily established among the sectors.

Taking also link directions and weights into account, the modularity *Q*_*d*, *w*_ as defined in Eq (19) is shown in Fig 4B for all partitions in the ITN for all years. Our previously described findings are further supported by the fact that the modularity *Q*_*d*, *w*_ is low for 𝓒_*s*_, whereas the values of *Q*_*d*, *w*_(𝓒_*c*_) are in the range of modularity values obtained with the community detection algorithm. Over the 1990s, we observe a decreasing trend of both *Q*_*d*, *w*_(𝓒_*c*_) and *Q*_*d*, *w*_(𝓒_*m*_), whereas the modularity of 𝓒_*s*_ is rising except for the period of the global financial crisis in 2009.

To assess the impact of the weights in the modularity calculation, Fig 4C shows the modularity *Q*_*d*_ by considering the degree and neglecting link weights in Eq (18). Here, the results show a decreasing trend in *Q*_*d*_ for all partitions. This decrease indicates an increasing entanglement of trade patterns—possibly due to a rising complexity, as partitions in trade patterns become less significant. In the ITN with constant link density for all years and neglecting weights (see Fig 4D) this trend for *Q*_*d*_ is considerably weaker. The qualitative differences between Fig 4B and 4C indicate that industries with large trade volumes contribute significantly to the value of *Q*_*d*, *w*_. From the comparatively high values of *Q*_*d*, *w*_ we can conclude that industries with large trade volume are grouped within tightly connected communities. The difference *Q*_*d*, *w*_(𝓒_*m*_) − *Q*_*d*, *w*_(𝓒_*c*_) increases slightly in Fig 4B. However, this difference does not exhibit marked changes over time when link weights are neglected (cf. Fig 4C and 4D).

To summarize the results presented above, our findings demonstrate that nations are still valid partitions in the sense of communities in complex network science. High trading industries build particularly tightly connected communities. However, the modularity shows a decreasing trend for all partitions in the ITN when link weights are neglected. This trend can be explained by new established links with comparatively low trade volume that cause a rising complexity of relationships within the global trade network. Interpreted in economic terms, these findings represent the increasing complexity in global supply chains.

### Role assignment in the ITN

The previously discussed partitions provide the basis for further analyses of the topological substructure of the ITN. Here the comparison between the internal topology of subgraphs and the cross-subgraph relations is of particular relevance. Certain nodes in the ITN often play a characteristic role in global supply chains. For example, some developing countries are specialized on the export of specific goods or resources. Thus, from the interacting network perspective, the respective industry stands out as a source of monetary flow across subgraphs in the national partition. To identify key industries and recognize their role in the global supply chain, we focus in the following on three network measures: node strength, (cross-)clustering coefficient and cross-betweenness.

#### Node strength

The strength of a node is a simple yet enlightening measure to quantify the importance of a node in the ITN, as it describes the total amount of monetary flow entering and leaving the node. The internal and cross-strength as defined in Eq (3) provide information about the trading partners of each node. In order to assess characteristic trade patterns of industries, *s*_*i*;auto_ and *s*_*i*;cross_ in partition 𝓒_*c*_ quantify the importance of an industry for domestic and international trade, respectively. Thus, we aggregate the strength values of industry *q* over all countries, $s_{q}=\sum_{i\in V_{{}^{{}_{q}}}^{\prime }}s_{i}$, for both the in-strength and out-strength. Table 2 summarizes the sectors with the highest trade volume in the ITN of 2005. We observe that financial services and business activities are particularly important for trade within a country, with domestic output amounting to 23.1% of global trade. This corresponds to a share of 28% of domestic trade as 81.9% of monetary flows in 2005 are transferred within the same country. The electrical and machinery industry holds the largest share of international trade, with $s_{q;\text{cross}}^{in}=$ 3.9%. Petroleum and chemical goods follow second in the ranking of cross-country trade.

**Table 2:** Key sectors for internal and cross-country trade in the ITN 2005.

**domestic input $s_{q;\text{auto}}^{in}$**		**domestic output $s_{q;\text{auto}}^{out}$**	
**%**	**industry**	**%**	**industry**
11.4	Financial Services & Businesses	23.1	Financial Services & Businesses
7.4	Electrical and Machinery	8.8	Petroleum, Chemical & Non-Metallic
7.1	Petroleum, Chemical & Non-Metallic	5.4	Transport
.	.	.	.
**foreign input $s_{q;\text{cross}}^{in}$**		**foreign output $s_{q;\text{cross}}^{out}$**	
**%**	**industry**	**%**	**industry**
3.9	Electrical and Machinery	2.9	Electrical and Machinery
3.2	Petroleum, Chemical & Non-Metallic	2.7	Petroleum, Chemical & Non-Metallic
1.6	Metal Products	1.4	Transport Equipment
.	.	.	.

**Fig 5:**
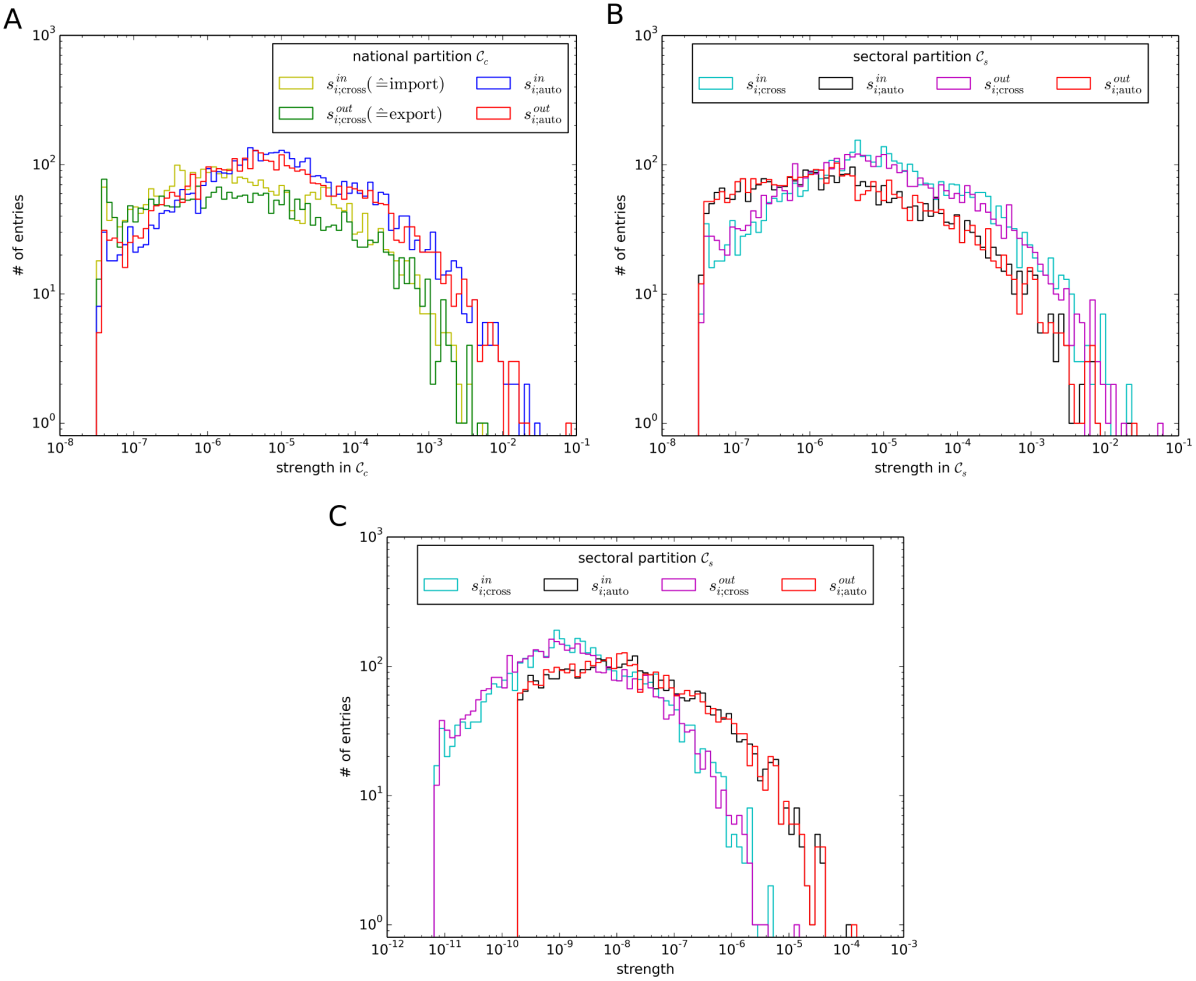
Distributions of in- and out-strength for the ITN in 2005. (A) According to the national partition 𝓒_*c*_ and (B) to the sectoral partition 𝓒_*s*_. (C) Average strength per potential trading partner.

Fig 5 shows the distributions of *s*_*i*;auto_ and *s*_*i*;cross_ for both the national partition 𝓒_*c*_ (A) and the sectoral partition 𝓒_*s*_ (B) for the year 2005. In 𝓒_*c*_ the distribution of domestic monetary flows is shifted towards higher values compared to cross-country flows. This indicates that domestic trade is likely to exceed international trade for randomly drawn nodes. As there are more sectors abroad than in the same country of a node, this statement is even strengthened in significance when flows per potential trading partners are considered. Again, this supports the viewpoint of national economies being interconnected subgraphs in the ITN. In the sectoral partition 𝓒_*s*_ cross-sectoral trade exceeds intra-sectoral trade by absolute value. However, taking into account that there are more extra-sectoral nodes than intra-sectoral ones, trade within the same sector dominates cross-sectoral trade by monetary flow per potential trading partner (see Fig 5C).

#### Clustering and cross-clustering coefficient

**Fig 6:**
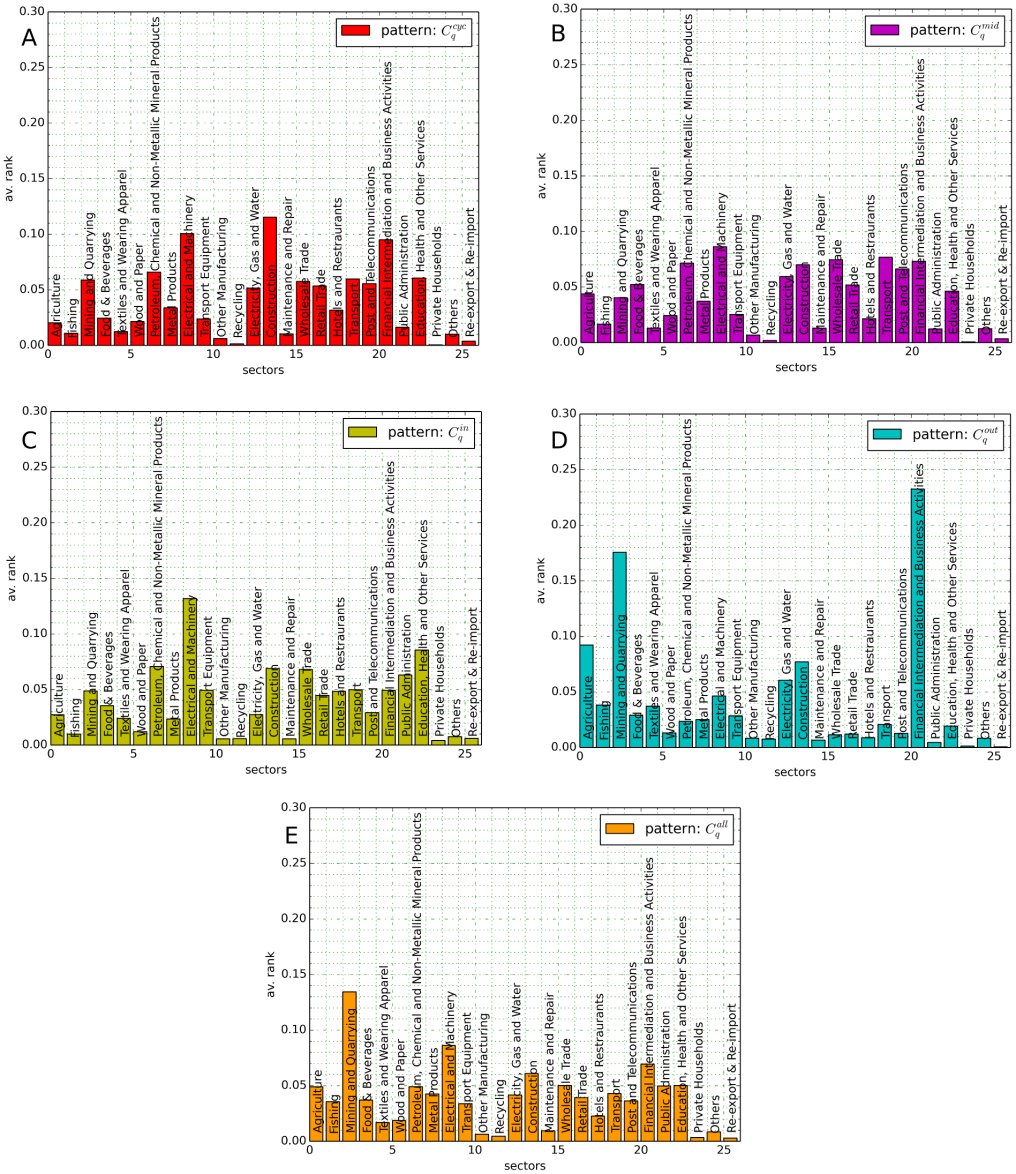
Average rank of clustering coefficients $C_{q}^{cyc}$ (A), $C_{q}^{mid}$ (B), $C_{q}^{in}$ (C), $C_{q}^{out}$ (D) and $C_{q}^{all}$ (E) as defined in Eq (21) for the ITN in 2005.

In the directed ITN the five definitions of clustering coefficients in Eq (4) describe different roles in the supply chain. We address the question to what extent industrial sectors show typical clustering patterns. Let $V_{{}^{{}_{q}}}^{\prime }$ be the subset of nodes belonging to sector *q* in 𝓒_*s*_ and $U_{{}^{{}_{i}}}^{\prime }$ be the subset of nodes in 𝓒_*c*_ belonging to the same country as node *i*. Then

$$C_{q}=\sum_{i\in V_{q}^{\prime }}\frac{C_{i}}{\sum_{j\in U_{i}^{\prime }}C_{j}}$$ (21)

is the sectoral mean value of the clustering coefficient *C* averaged over all countries. In order to avoid that the properties of the major economies dominate the results, Eq (21) is normalized such that countries with high trade volume equally contribute to the average as countries with few trade. Fig 6 illustrates the results for the clustering coefficients as defined in Eq (21) in the year 2005.

One observes characteristic distributions for the different clustering coefficients. In particular, the motif $C_{q}^{out}$ appears comparatively more frequent in nodes belonging to financial intermediation & business activities (cf. Fig 6D). This underlines the importance of the financial industry as capital provider for investments. Raw materials and resources are produced in mining & quarrying industries and are often subsequently sold to other sectors, leading to a high rank in $C_{q}^{out}$. As shown in Fig 6B, the motif $C_{q}^{mid}$ is frequently observed for sectors related to trade and such that produce secondary products (e.g. petroleum, machinery). The construction industry is dominant in the motif $C_{q}^{cyc}$ (cf. Fig 6A), whereas electrical and machinery industries dominate the pattern of $C_{q}^{in}$ (cf. Fig 6C).

**Fig 7:**
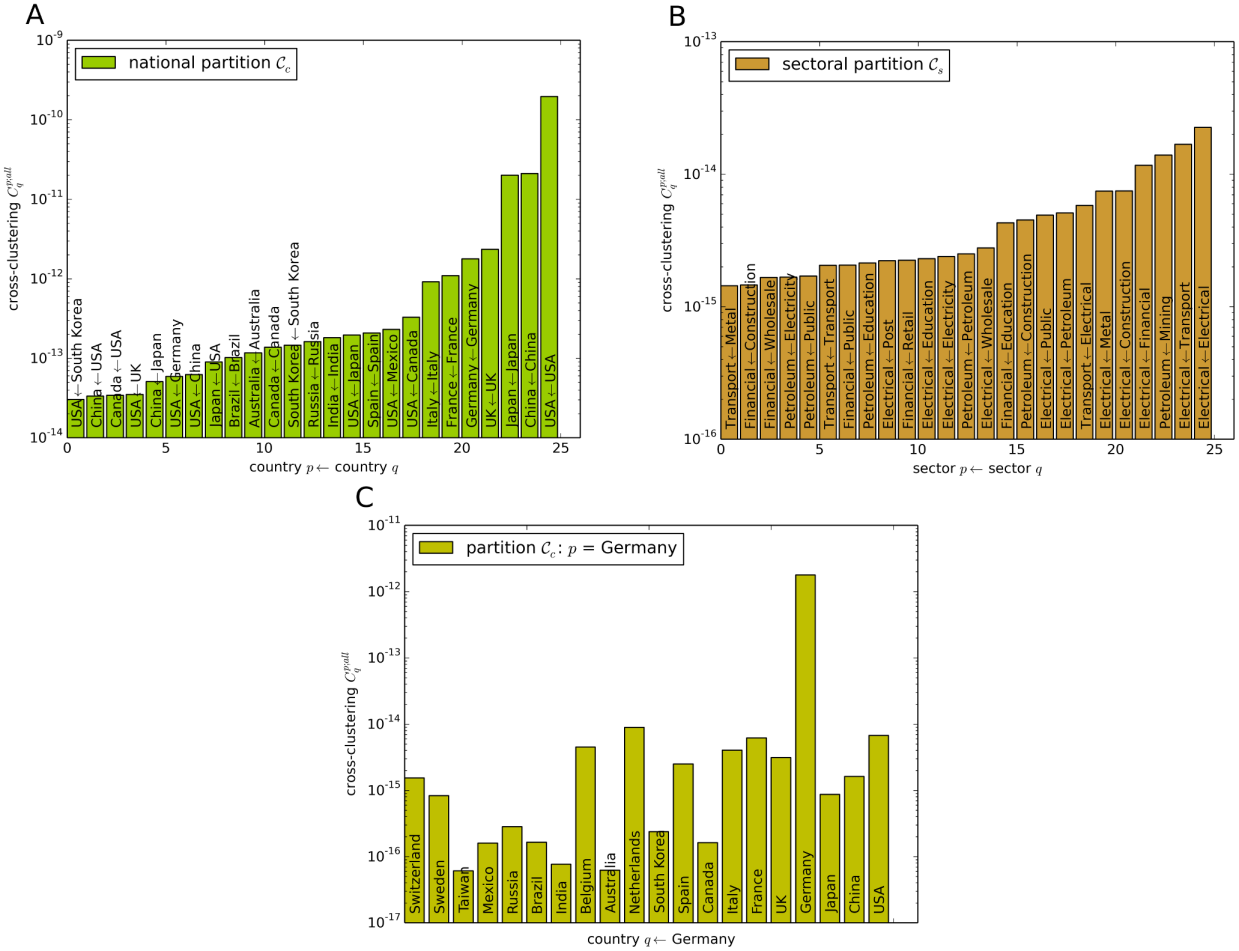
Ranking of the global cross-clustering coefficient. $C_{q}^{p;all}$ for pairs of countries (A) and sectors (B). (C) As an example, the global cross-clustering coefficients between Germany and the world’s largest economies are shown.

The global cross-clustering coefficient as defined in Eq (9) sheds light on characteristic trade patterns between subgraphs in the world trade network. We measure $C_{q}^{p;all}$ for all combinations of *p* and *q* in the national partition 𝓒_*c*_ and the sectoral partition 𝓒_*s*_. A summary of the highest obtained values is presented in Fig 7A and 7B. In the national partition, the cross-clustering coefficient $C_{p}^{q}$ is highest if *p* = *q* for the world’s largest economies. This is a reasonable behavior, as we have observed a high link density and trade volume in these national economies. Similar results are obtained for other directionality patterns of the clustering coefficient. As internal trade volume in subgraph *p* enters through a factor in the calculation of $C_{i}^{p}$ (see Eq (6)), subgraphs with a large trade volume exhibit large global cross-clustering coefficients. Therefore, the USA are involved in 20 of the 30 top global cross-clustering values in 𝓒_*c*_ (cf. Fig 7A). Furthermore, we conclude that the global cross-clustering coefficient is large between countries with high trade volumes and short geographical distance. For example, industries in Canada and Mexico score a high cross-clustering coefficient in the USA and the Netherlands, Belgium and France score high values in Germany (cf. Fig 7C). In 𝓒_*s*_ the electrical and machinery industry is the dominant sector (cf. Fig 7B).

#### Cross-Betweenness

By definition, the betweenness of a node provides an estimate of a sector’s importance in the global trade network. A higher resolved picture is provided by the cross-betweenness (Eq (7)) that is confined to geodesics between two subgraphs. In particular, the values $b_{i}^{pq}$ of nodes not belonging to either *p* and *q* contain vital information about the node’s importance in connecting these subgraphs. For each pair (*p*, *q*) of the 30 countries with the highest trade volume in 𝓒_*c*_ (26 industry sectors in 𝓒_*s*_), we calculate the cross-betweenness fraction from nodes belonging to a third subgraph:

$$\beta^{pq}=\sum_{i\notin V_{p}^{\prime }\cup V_{q}^{\prime }}b_{i}^{pq}/\sum_{j\in V}b_{j}^{pq}\;.$$ (22)

**Fig 8:**
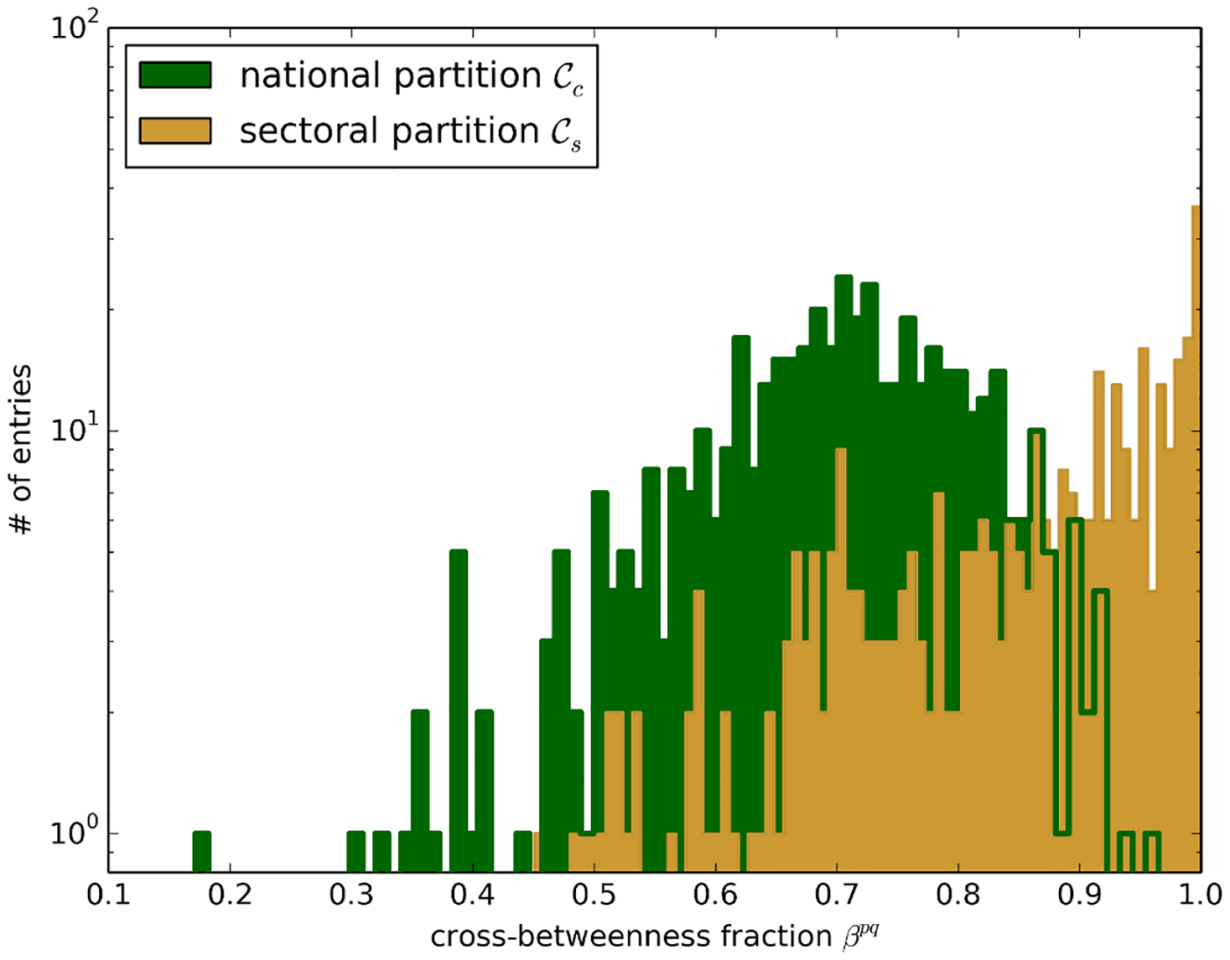
Distribution of cross-betweenness fraction *β* (Eq (22)) for pairs in the national partition 𝓒_*c*_ and the sectoral partition 𝓒_*s*_ of the ITN in the year 2005.

A low value of *β* ^*pq*^ implies strong direct relations between subgraphs *p* and *q* as most geodesics from nodes in *p* to nodes in *q* do not cross a third subgraph. The distributions of *β* ^*pq*^ in the national and sectoral partition are shown in Fig 8.

We observe that the distribution of *β* ^*pq*^ for the national partition is shifted towards lower values compared to 𝓒_*s*_. This is another indicator of the strong connectivity within national economies, as shortest paths between two countries often do not cross an additional third country. In fact, in the sectoral partition *β* peaks at about 1. Thus, there are many shortest paths from sector *p* to sector *q* that run through at least one additional industry sector.

We are interested in identifying the countries and industries that play a significant role in connecting subgraphs *q* and *p*. Thus, we calculate the average over pairs (*p*, *q*) taking only values $b_{i}^{pq}$ into account if $i\notin V_{{}^{{}_{p}}}^{\prime }\cup V_{{}^{{}_{p}}}^{\prime }$:

$$\langle b_{i}\rangle =\underset{p<q}{\sum }\frac{b_{i}^{pq}\left(1-\delta_{ip}\right)\left(1-\delta_{iq}\right)}{\sum_{j\notin V_{p}^{\prime }\cup^{}V_{q}^{\prime }}b_{j}^{pq}}/\left(\begin{array}{c} n_{q}\\ 2 \end{array}\right)\;.$$ (23)

Here, *δ*_*ip*_ is defined as 1 if $i\in V_{{}^{{}_{p}}}^{\prime }$, and 0 otherwise. The number of subgraphs that are considered in the average is represented by *n*_*q*_, normalizing Eq (23) such that $\Sigma_{i=1}^{N}\langle b_{i}\rangle =1$. The importance of subgraph *p* in connecting other subgraphs is then obtained by $\sum_{i\in V_{{}^{{}_{p}}}^{\prime }}\langle b_{i}\rangle$. Our results show that for the national partition 𝓒_*c*_, Germany, USA and Switzerland hold most geodesics (Table 3). In the sectoral partition, however, USA, Germany and China lead the list with the financial services & business activities being the sector with largest 〈*b_i_*〉 (Table 4).

**Table 3:** Average cross-betweenness as defined in Eq (23), aggregated by country.

**National partition 𝓒_*c*_**		**Sectoral partition 𝓒_*s*_**	
∑ 〈***b**_i_*〉	**country**	∑ 〈***b**_i_*〉	**country**
0.145	Germany	0.114	USA
0.103	USA	0.074	Germany
0.089	Switzerland	0.064	China
0.079	UK	0.038	France
0.066	China	0.036	Netherlands
0.061	Netherlands	0.032	Italy
0.050	Japan	0.030	Belgium
0.049	Italy	0.029	UK
0.043	France	0.025	Japan
0.041	Belgium	0.020	South Africa

**Table 4:** Average cross-betweenness as defined in Eq (23), aggregated by industry.

**National partition 𝓒_*c*_**		**Sectoral partition 𝓒_*s*_**	
∑ 〈***b**_i_*〉	**industries**	∑ 〈***b**_i_*〉	**industries**
0.178	Re-export & Re-import	0.193	Finance & Business
0.153	Petroleum	0.146	Petroleum
0.152	Finance & Business	0.115	Electrical and Machinery
0.119	Electrical and Machinery	0.107	Re-export & Re-import
0.060	Metal Products	0.098	Transport
0.047	Transport	0.060	Food & Beverages
0.043	Wood and Paper	0.045	Metal Products
0.038	Education	0.031	Education
0.035	Food & Beverages	0.027	Mining and Quarrying
0.028	Textiles	0.025	Agriculture

#### Statistical interdependencies between local network measures

A priori it is not known how the different measures introduced above contribute to complementary information about the network’s topology. In order to assess this issue, we investigate potential statistical interdependencies between cross-node strength, cross-clustering coefficient and cross-betweenness and present illustrative examples. A further theoretical study about possible correlations between the introduced network measures is beyond the scope of this work.

**Fig 9:**
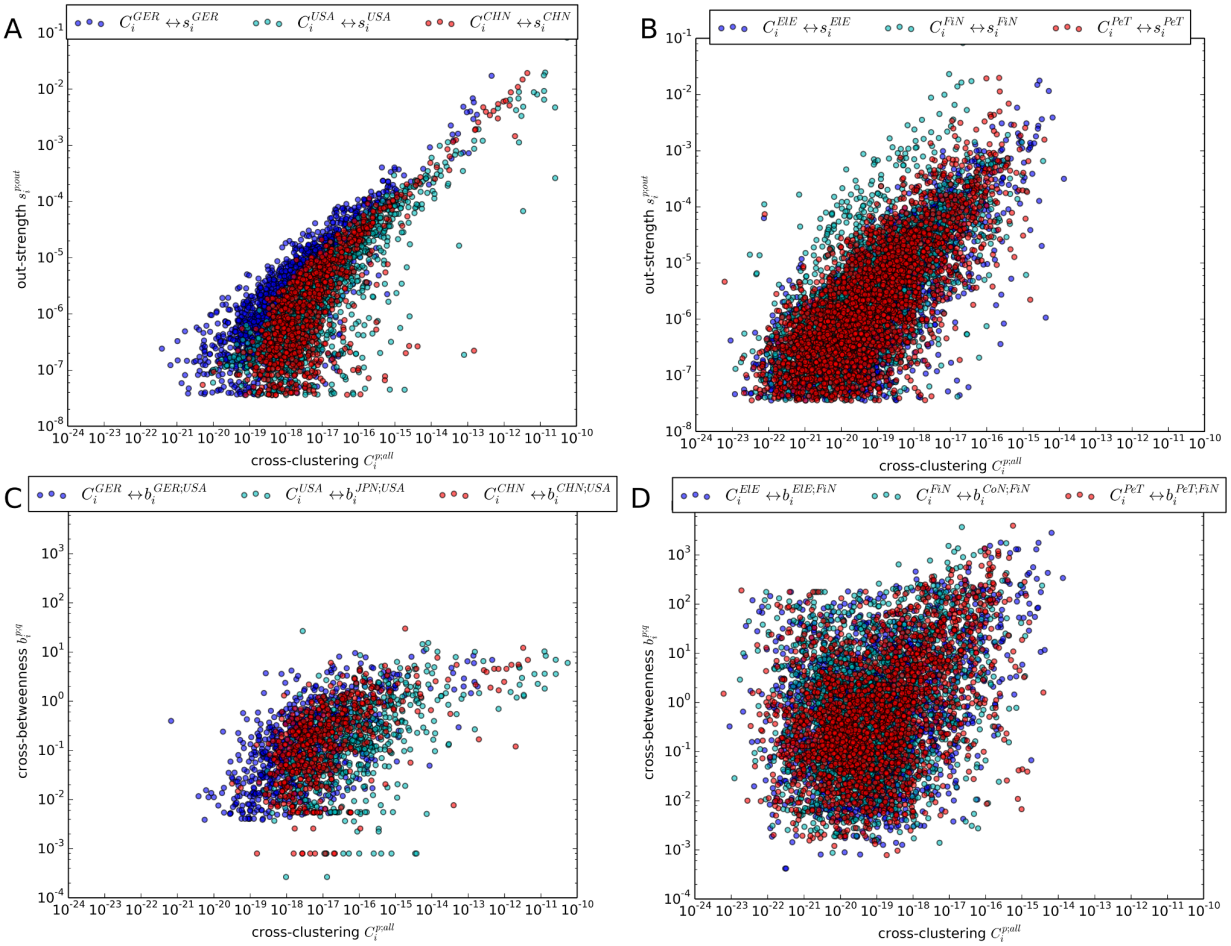
Correlation between network measures for different subgraphs (*p*, *q*). The scatter plots depict: (A) the local cross-clustering coefficient $C_{i}^{p;all}$ and the cross-strength $s_{i}^{p;out}$ for subgraphs in the national partition: China [CHN], Germany [GER] and USA. (B) $C_{i}^{p;all}$ and $s_{i}^{p;out}$ for the sectoral partition: Petroleum & Non-metallic products [PeT], Finance & Businesses [FiN], and Electrical & Machinery [ElE]. (C) $C_{i}^{p;all}$ and the cross-betweenness $b_{i}^{p;q}$ in the national partition: Japan [JPN], Germany, China and USA. (D) $C_{i}^{p;all}$ and $b_{i}^{p;q}$ in the sectoral partition: PeT, FiN, ElE and Construction [CoN].

As link weights enter directly the calculation of the local cross-clustering coefficient in Eq (4), we consider the correlation between $C_{i}^{p;all}$ and the monetary flow $s_{i}^{p;out}:=\underset{j\in V_{p}^{\prime }}{\sum }w_{ij}$ from node *i* into subgraph $V_{p}^{\prime }$. From the scatter plots we observe a stronger and generally positive statistical relationship between both characteristics in the national partition (cf. Fig 9A) than in the sectoral partition (cf. Fig 9B). The scatter plot between the local cross-clustering coefficient and the cross-betweenness exhibits a similar picture. In the national partition, shown in Fig 9C, the two measures show a stronger interdependence than in the sectoral partition in Fig 9D. This is due to the fact, that in the sectoral partition, one subgraph consists of 186 nodes from countries with very different economic performances, leading to a wide spread of $C_{i}^{p;all}$. However, the national partition exhibits fewer variability in the strengths of the 26 nodes that belong to the same country. We conclude that the cross-strength (measuring the overall monetary flow originating from a node), the local cross-clustering coefficient (quantifying the occurrence of motifs across subgraphs), and the cross-betweenness (characterizing a node’s importance in connecting two subgraphs) capture different aspects of a node’s role in the ITN, although these three concepts are not fully unrelated conceptually.

### Evolution of global interacting network measures in the ITN

As a final aspect, we study the evolution of the ITN and how globalization is represented in the topological properties and substructures of global trade. As already demonstrated by the decreasing trend of modularity (see Fig 4B), the community structure in the network has become less significant along with the process of globalization. In the following we discuss how reorganization of trade patterns affects the network structure at both the local (node strength) and global scale (link density, reciprocity). We further investigate the speed of the reorganization process via the Hamming distance and discuss relevant measures to observe anomalies in trade patterns, in particular economic crises.

#### Node strength

**Fig 10:**
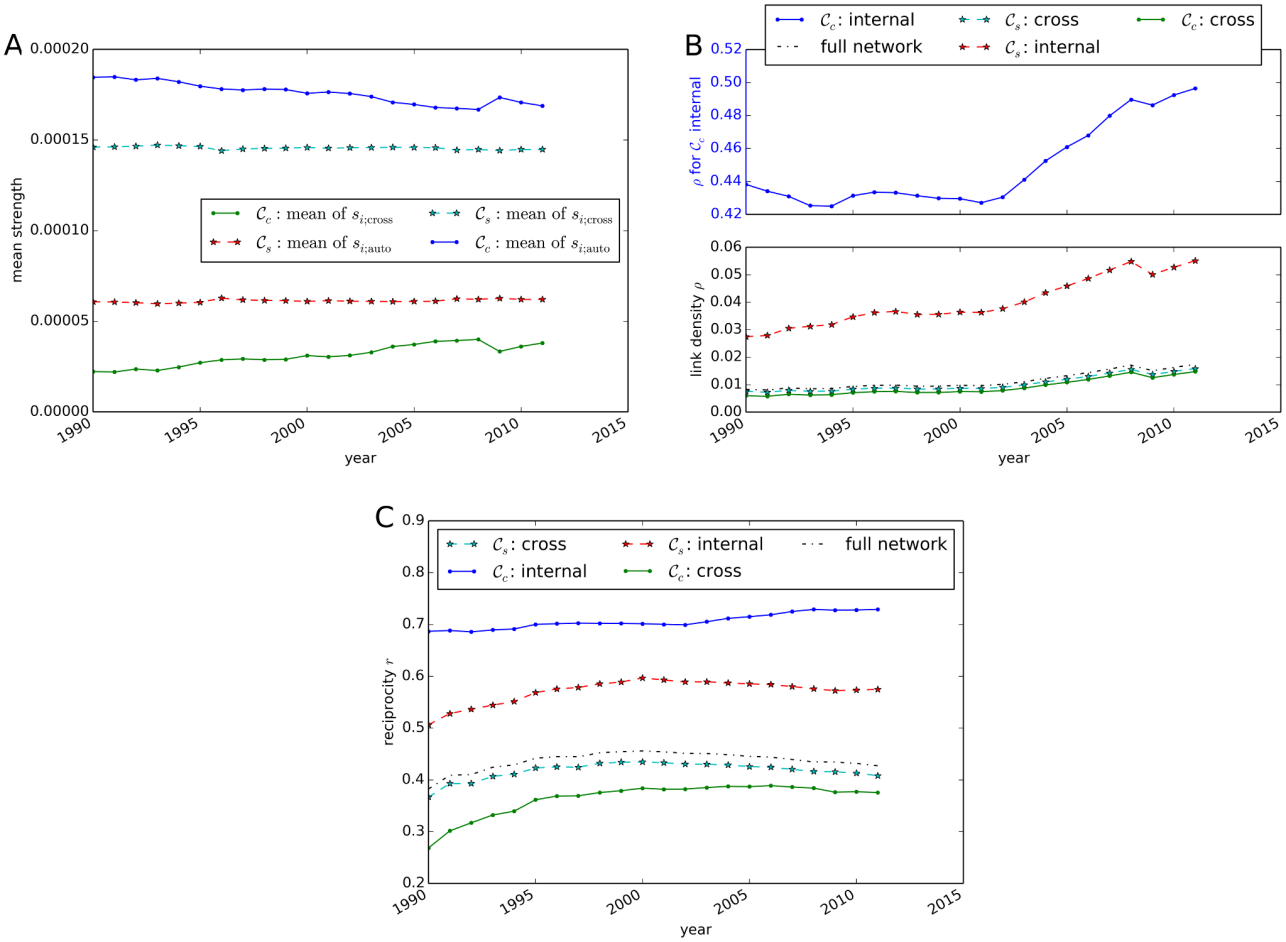
Evolution of ITN characteristics between 1990 and 2011. (A) Mean node strength, (B) link density and (C) reciprocity. In addition to the properties of the full network, internal and cross-measures for the sectoral (𝓒_*s*_) and national (𝓒_*c*_) partition are shown.

In order to assess the evolution of the strength distributions presented in Fig 5 we calculate the mean for each year between 1990 and 2011. Due to the fact that all monetary flows are contained within the network, the mean of the output and input distributions is identical. The employed normalization process to avoid inflationary effects during network construction implies opposing trends in the means of *s*_*i*;auto_ and *s*_*i*;cross_. In Fig 10A a trend towards more international trade can be observed from the evolution of the means in the national partition. However, in 2011 the mean domestic strength is still 4.4 times higher than the respective value for international relations. In the sectoral partition no comparable trend is observed with a practically stable mean. One could expect that technological progress leads to an adaptation of production functions to new technologies, and thus results in an adjustment of input requirements. However, in the classification of industry sectors these effects are small compared to the observed changes in the national partition.

#### Link density and reciprocity

Fig 10B reveals an increasing trend in the link density in all parts of the network. The internal link density *ρ*_auto_ for domestic trade reaches 50% in 2011, whereas the density for the full network *ρ* accounts for only 1.7%. In the sectoral partition, *ρ*_auto_ exceeds *ρ*_cross_ with max(*ρ*_auto_) = 5.5% in 2011. The results might be slightly biased due to the fact, that data from national bureaus of statistics serve as main sources for the construction of the Eora MRIO database, leading potentially to more accurate national data compared to international monetary flows [18]. However, this bias is not able to explain the observed magnitude of differences in the link density between national and international trade. Therefore, our results further emphasize the importance of trade relations within national economies. A deviation from the trend of increasing link density is observed in 2009, when the link density decreased compared to the previous year. This effect coincides with the financial crisis in 2008/2009 that caused many countries to experience a recession in 2009 [49].

The reciprocity (Fig 10C) exhibits a different behavior depending on the considered partition. While *r*_auto_ gradually increases in the national partition 𝓒_*c*_, the reciprocity value peaks for the full network in the year 2000. This indicates that domestically, new links are mainly established between sectors that already possess a one-way trade relationship. However, reciprocity in cross-country relations saturates in 2000. For the full network and in the sectoral partition 𝓒_*s*_, reciprocity even decreases after 2000. This indicates, that in this period most emerging links are added as new one-way trade relationships between industrial sectors.

#### Hamming distance

**Fig 11:**
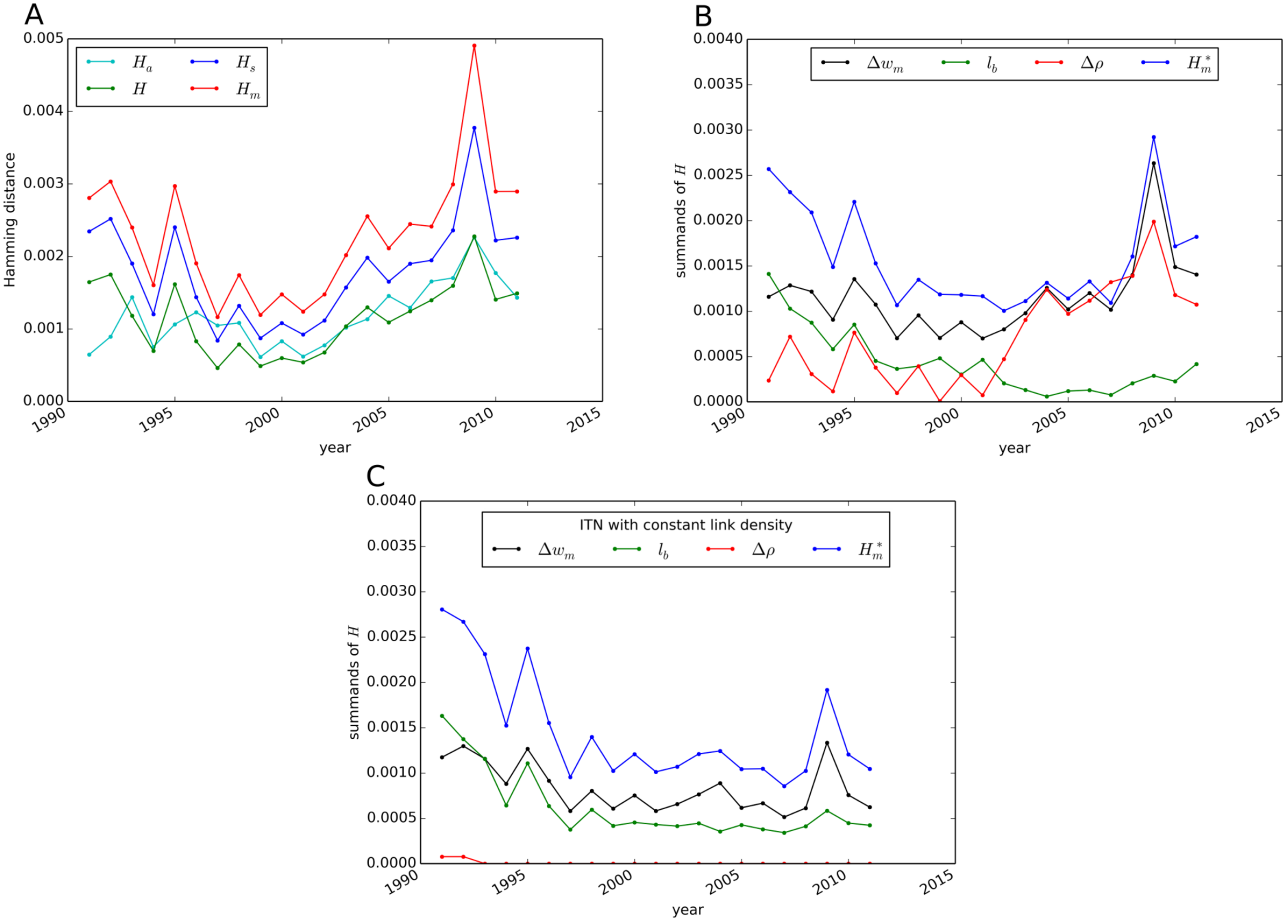
Hamming distance between the ITN in the current and the respective preceding year for 1991–2011. Various generalizations of *H* to weighted networks are compared (A). The contributions as defined in Eq (16) are illustrated for the ITN constructed with thresholding (B) and constant link density (C).

To quantify the restructuring of trade relations, we measure the Hamming distance between the ITN in the present and the preceding year. First, we compare the results of the different generalizations (Eq (15)) of the Hamming distance (see Fig 11A). The graphs of *H*_*m*_, *H*_*s*_ and *H* follow identical trends with *H*_*m*_ peaking in 2009 at a value of 0.0049. To better understand the underlying dynamics of the reorganization process, we measure the decomposition of the Hamming distance as defined in Eq (16). We observe an increasing effect of link density differences since the year 2000 (see Fig 11B). In the corrected Hamming distance $H_{m}^{*}$ the rising trend since 2000 is significantly reduced. Therefore, $H_{m}^{*}$ is an applicable measure to identify anomalies in trade patterns, such as the financial crisis in 2009. Comparatively large values and fluctuations are visible in the early 1990s. These can partly be explained by an adaptation of trade pattern to the new global political and economic landscapes that arose after the collapse of the Soviet Union in 1991. Furthermore, the contribution of the difference in weights Δ*w*_*m*_ to the Hamming distance increases compared to the blinking links *l*_*b*_. For comparison, Fig 11C shows the Hamming distance in the ITN with constant link density for each year. In this network, the absolute values of $H_{m}^{*}$ are lower than in the threshold based construction of the ITN. The peak in 2009 is still visible in the network with constant link density, although less significant compared to the reorganization in the 1990s. We also measure the Hamming distances restricted to internal and cross-subgraph connections for 𝓒_*c*_ and 𝓒_*s*_. However, differences in the trends are comparatively small, which implies that reorganization in trading patterns occurs in both internal and cross-subgraph relations.

## Discussion

In this paper we have shown that the ITN, interpreted as a network of interdependent subnetworks, exhibits a non-trivial and dynamic architecture. The methodology and tools presented are well-suited for the assessment of both global and local properties of this network. Our study provides a profound basis and reference for addressing more detailed research questions and case studies on trade networks in the future. One of these studies, for example, could include an assessment of impacts of specific trade agreements between regions on the structure of global trade. The cross-betweenness would serve here as an appropriate tool in order to quantify possible changes of a subnetwork’s importance in the global supply chain.

We have addressed the question how both national economies and the sectoral partition stand out in the global network that consists of highly interwoven trade relationships. Our results demonstrate that the country-based partition of industrial sectors closely resembles the notion of communities in complex networks. However, an even higher modularity is achieved with a suitable community detection algorithm, pointing to an increasing relevance of international trade relationships. Important factors like geographical proximity and political linkages between countries are expressed in the observed community structure. A more detailed assessment of these factors and their implications for the network structure is an interesting subject for further study. Clusters in the sectoral partition do not exhibit the characteristic linkage features of communities. Nevertheless, by observing trade patterns in the sectoral partition, new insights into the structure of the network are obtained.

A second key aspect of this study is the assignment of roles to the nodes in the ITN. Having defined meaningful partitions, the distinction between internal and cross-subgraph properties provides a new tool for unveiling the core functions of different sectors. For example, we find that domestic trade is dominated by the financial services & business activities and that the trade activity of this sector accounts for > 20% of global output. The clustering coefficient allows to assess directionality patterns and to find characteristic roles of sectors in the supply chain. Among others, the mining sector is identified as a predominantly output producing industry, whereas trade businesses appear more frequently in the center of global supply chains. Pairs of countries that are geographically close or exhibit large trade volume are often characterized by high cross-clustering coefficients. Further more detailed insights into the functional roles of industrial sectors and countries are provided by the cross-betweenness.

Finally, we have illustrated how globalization and economic crises have manifested themselves in the evolution of the substructure of the ITN. The increase of international interdependence is well observed in global network measures such as modularity and link density. The almost continuous decrease in unweighted modularity for both the optimal and the national partition suggests an overall increase in the complexity of trade relations, where the partition into national economies becomes less significant. However, the trends of most relevant network measures are interrupted in the year 2009, which coincides with the consequences of the global financial and economic crisis. We have successfully introduced a meaningful generalization of the Hamming distance to weighted networks that serves as a good indicator for the associated strong reorganization processes of trade patterns.

In order to further strengthen the interpretations of our analysis, future work requires a sophisticated mapping of monetary flows to the trade of goods in terms of physical material flows. The price evolutions of different goods are highly heterogeneous and volatile on short time-scales, leading to noise in the absolute values of monetary flows. Consequently, changes in monetary values do not always represent changes in the physical flows of goods [33]. In order to minimize artifacts originating from price fluctuations, a comparison of the results with databases summarizing physical merchandise trade flows between industries would be required. Another opportunity for more detailed research is associated with community detection in the directed ITN. Although a number of suggestions for a generalized quality function of partitions have been formulated for directed networks (e.g. [46, 50]), the intuition of a partition with high inter-cluster and low internal link density is not as straightforward as in an undirected network [43]. The assessment of different quality functions and algorithms that take the directionality pattern in an economically meaningful way into account would refine the results of this study. We outline corresponding detailed analyses as a subject of future work.
